# A Comparison of SARC-F, Calf Circumference, and Their Combination for Sarcopenia Screening among Patients Undergoing Peritoneal Dialysis

**DOI:** 10.3390/nu14050923

**Published:** 2022-02-22

**Authors:** Yu-Li Lin, Chih-Hsien Wang, Jen-Pi Tsai, Chih-Tsung Chen, Yi-Hsin Chen, Szu-Chun Hung, Bang-Gee Hsu

**Affiliations:** 1 Division of Nephrology, Hualien Tzu Chi Hospital, Buddhist Tzu Chi Medical Foundation, Hualien 97004, Taiwan; nomo8931126@gmail.com (Y.-L.L.); wangch33@gmail.com (C.-H.W.); 2 School of Medicine, Tzu Chi University, Hualien 97004, Taiwan; tsaininimd1491@gmail.com (J.-P.T.); nephp06@gmail.com (Y.-H.C.); szuchun.hung@gmail.com (S.-C.H.); 3 Division of Nephrology, Department of Internal Medicine, Dalin Tzu Chi Hospital, Buddhist Tzu Chi Medical Foundation, Chiayi 62247, Taiwan; 4 Division of Nephrology, Department of Internal Medicine, Taichung Tzu Chi Hospital, Buddhist Tzu Chi Medical Foundation, Taichung 40201, Taiwan; a0983262273@gmail.com; 5 Division of Nephrology, Department of Internal Medicine, Taipei Tzu Chi Hospital, Buddhist Tzu Chi Medical Foundation, Taipei 23142, Taiwan

**Keywords:** SARC-F, SARC-CalF, calf circumference, sarcopenia, peritoneal dialysis

## Abstract

Sarcopenia is frequently encountered in patients undergoing peritoneal dialysis (PD). We evaluated and compared the diagnostic performance of a strength, assistance walking, rise from a chair, climb stairs, and falls (SARC-F) questionnaire, SARC-F combined with calf circumference (SARC-CalF), and calf circumference (CC) for screening sarcopenia among patients undergoing PD. We measured the appendicular skeletal muscle mass, evaluated using a multifrequency bioimpedance spectroscopy device, handgrip strength, and 6-m gait speed. SARC-F, SARC-CalF, and CC were obtained in all participants. Sarcopenia was defined using four different diagnostic criteria, including the Asian Working Group for Sarcopenia (AWGS) 2019, revised European Working Group on Sarcopenia in Older People (EWGSOP2), Foundation for the National Institutes of Health (FNIH), and International Working Group on Sarcopenia (IWGS). Among 186 enrolled patients undergoing PD (mean age 57.5 ± 14.1 years), the sarcopenia prevalence was 25.8–38.2% using the four definitions. The discriminative powers of SARC-CalF (range 0.648–0.748) and CC (range 0.652–0.813) against the four definitions were better than those exhibited by SARC-F (range 0.587–0.625), which achieved significant difference, except when adopting the criteria of the FNIH. After stratification by gender, the superiority of SARC-CalF and CC over SARC-F was maintained when AWGS 2019, EWGSOP2, and IWGS were applied. In conclusion, CC and SARC-CalF outperformed SARC-F in the diagnostic accuracy of sarcopenia among patients undergoing PD.

## 1. Introduction

Sarcopenia, characterized by an aging-related progressive decline of skeletal muscle mass, strength, and physical performance, is associated with adverse clinical outcomes [1,2,3]. As chronic kidney disease progresses, the accelerated muscle wasting resulting from multifactorial and intricate pathogenesis accounts for the considerably high prevalence of sarcopenia when patients reach end-stage renal disease (ESRD) [4]. In particular, patients undergoing peritoneal dialysis (PD) have substantial daily losses of protein during the process of dialysis [5]. Therefore, it is crucial to develop feasible and easy-to-use screening tools facilitating the rapid detection of sarcopenia among patients undergoing PD in an attempt to provide timely therapeutic intervention.

The strength, assistance walking, rise from a chair, climb stairs, and falls (SARC-F), a five-item self-reported questionnaire first developed in 2013 [6], is a well-established and widely used initial screening tool for geriatric sarcopenia and has been recently recommended by the Asian Working Group for Sarcopenia (AWGS) 2019 and the revised European Working Group on Sarcopenia in Older People (EWGSOP2) [7,8]. Moreover, calf circumference (CC) has been used to screen geriatric sarcopenia, which provided moderate-to-high sensitivity and specificity in the Asian population [9,10,11]. Accordingly, the AWGS 2019 also recommends screening sarcopenia using CC and SARC-F combined with calf circumference (SARC-CalF) [8], the latter of which adds CC item into the original SARC-F scale [12].

There is increasing evidence suggesting an improved diagnostic accuracy and sensitivity of SARC-CalF compared with the original SARC-F version in the geriatric and cancer population [12,13,14,15]. However, only a few studies have addressed the clinical utility of SARC-F, CC, and SARC-CalF among the dialysis population. Although the use of SARC-F among patients undergoing PD has been recently reported [16], whether CC and SARC-CalF are superior to SARC-F in the diagnostic performance of sarcopenia in this vulnerable population remains unexplored.

Hence, this study was conducted to evaluate and compare the diagnostic performance of SARC-F, SARC-CalF, and CC in sarcopenia screening among patients undergoing PD.

## 2. Materials and Methods

### 2.1. Study Design and Participants

This cross-sectional study was conducted at Hualien Tzu Chi Medical Center and its three branch hospitals at Dalin, Taichung, and Taipei during the period between February 2020 and May 2021. All patients aged > 20 years who underwent PD for more than 3 months were invited to participate in the study. Exclusion criteria were acute infection, active malignancy, a pacemaker or defibrillator, amputated limb(s), bedridden status, and those who refused to participate.

Basic demographic data, PD duration and modality, and medical histories, including diabetes mellitus (DM), hypertension, and hyperlipidemia, were collected through electronic medical records. Smoking status was obtained through interviews. This study was approved by the Institutional Review Board of Tzu Chi Hospital (IRB 108-219-A), and all participants provided written informed consent according to the general recommendations of the Declaration of Helsinki.

### 2.2. Anthropometric and Skeletal Muscle Measurements

Height was measured without shoes, and body weight was measured with the participants wearing light clothing. Body mass index (BMI) was calculated as body weight (kg) divided by height squared (m^2^). In the erect standing position, waist circumference (WC) was measured at the shortest point between the lower rib margin and the iliac crest; mid-arm circumference (MAC) and triceps skinfold (TSF) were measured at the midpoint between the acromion and olecranon using a skinfold caliper (QuickMedical, Issaquah, WA, USA) and a flexible inextensible tape, respectively. The average value of the three TSF readings was accepted, and the mid-arm muscular circumference (MAMC) was subsequently calculated as MAC (cm) − π × TSF (cm). In a sitting position with the knee and ankle at a right angle, CC was measured at the point of greatest circumference on both legs using a nonelastic but flexible plastic tape, and average values were adopted for further analysis.

In a supine position, skeletal muscle and fat tissue mass were evaluated using a portable whole body multifrequency bioimpedance spectroscopy device (BCM, Fresenius Medical Care, Bad Homburg, Germany), which measures impedance spectroscopy at 50 frequencies. The BCM has been widely used to evaluate body composition in patients undergoing dialysis, whose measurement is less affected by hydration status [17,18,19]. Appendicular skeletal muscle mass (ASM) was derived from the following equation: ASM (kg) = −1.838 + 0.395 × total body water (L) + 0.105 × body weight (kg) + 1.231 × male sex − 0.026 × age (years). The ASM derived from the well-validated equation exhibited a value of R^2^ = 0.914, compared with the ASM measured by dual-energy X-ray absorptiometry in a Taiwanese dialysis cohort [20]. Appendicular skeletal muscle index (ASMI) and fat tissue index (FTI) were calculated as ASM (kg) and fat tissue mass (kg) divided by height squared (m^2^), respectively.

Handgrip strength (HGS) was measured using a handheld dynamometer (Jamar Plus Digital Hand Dynamometer, SI Instruments Pty Ltd., Hilton, Australia). In the standing position, patients were instructed to grip the dynamometer as tightly as possible, with the arm at a right angle and the elbow at the side of the body. Three measurements were repeated in each hand, with a 1-min rest interval. The average value of both hands was adopted for analysis.

For the usual gait speed (GS) measurement, patients were instructed to walk at their usual speed for 6 m on a flat and straight path, and the speed was calculated accordingly. The GS test was not performed on 18 patients with difficulty walking, and they are classified as having slow GS.

### 2.3. Definition of Sarcopenia

Four different definitions of sarcopenia based on geriatric consensus panels were adopted simultaneously in our study, including the AWGS 2019 [8], EWGSOP2 [7], Foundation for the National Institutes of Health (FNIH) [21], and International Working Group on Sarcopenia (IWGS) [22]. The diagnostic criteria and cut-off points to define sarcopenia from the consensuses are summarized in Table 1.

### 2.4. SARC-F and SARC-CalF

SARC-F includes the following five self-reported domains: strength, assistance with walking, rise from a chair, climb stairs, and falls. Three severity levels ranging from 0 (no difficulty or no fall) to 2 (great difficulty or more than four falls) were assigned to each component. The total score ranges between 0 and 10 [6]. For the scoring of SARC-CalF, a CC item, which was scored as 10 points if the CC is <34 cm for males and <33 cm for females, was added to the original SARC-F score [12]. 

### 2.5. Subjective Global Assessment

The subjective global assessment (SGA) consists of the following seven domains: weight change, dietary intake, gastrointestinal symptoms, functional capacity, comorbidity, subcutaneous fat, and signs of muscle wasting. A score ranging from 1 (normal) to 5 (very severe) was assigned to each component, and the sum score of all seven components ranged between 7 (normal) and 35 (severely malnourished) [23].

### 2.6. Laboratory Data

Fasting blood samples (~5 mL) were collected. After determining the blood cell count using approximately 0.5 mL of blood samples (Sysmex SP-1000i, Sysmex American, Mundelein, IL, USA), the remaining volumes were immediately centrifuged for biochemical analysis. An autoanalyzer (Siemens Advia 1800, Siemens Healthcare GmbH, Erlangen, Germany) was used to measure the serum concentrations of blood urea nitrogen (BUN), creatinine (Cr), albumin, and phosphorus. Intact parathyroid hormone (PTH) level was measured using enzyme-linked immunosorbent assays (Diagnostic Systems Laboratories, Webster, TX, USA). The 24-h urine and dialysate samples were collected for calculating the fractional clearance index for urea (Kt/V) using standard methods [24]. The total Cr excretion was calculated as the sum of 24-h creatinine excretion from urine and dialysate. Daily protein intake was estimated as the normalized protein nitrogen appearance rate (nPNA) [25].

### 2.7. Statistical Analysis

Assuming that the areas under curves (AUCs) of 0.70 for the screening tools are significant from the null hypothesis value 0.50, with a ratio of 3:1 between negative and positive cases in each gender [16], a total of at least 176 patients should be enrolled to achieve a power of 80% (α-level 0.05).

Continuous variables were expressed as mean ± standard deviation or median (interquartile range) based on the normality of the variables tested using the Kolmogorov–Smirnov test, and comparisons between genders were performed using the independent *t*-test or the Mann-Whitney U test. Categorical variables were expressed as absolute (*n*) and relative frequency (%) and compared using the χ^2^ or Fisher’s exact test, as appropriate.

The correlations of SARC-F, SARC-CalF, and CC with anthropometric and skeletal muscle measurements were analyzed using Pearson’s or Spearman’s rank correlation coefficient, according to the normality of variables. Receiver operating characteristic curves were generated to evaluate the diagnostic values of SARC-F, SARC-CalF, and CC on sarcopenia. The AUCs, cut-off values, sensitivity, specificity, positive predictive value (PPV), and negative predictive value (NPV) were established. The DeLong test was adopted for the paired comparisons of discriminative powers among the three screening tools [26]. 

Data were analyzed using SPSS for Windows (version 19.0, IBM Corp., Armonk, NY, USA) and MedCalc Statistical Software version 18.2.1 (MedCalc Software bvba, Ostend, Belgium). A value of *p* < 0.05 was considered to be statistically significant.

## 3. Results

A total of 186 patients undergoing PD, with a mean age of 57.5 ± 14.1 years and a median PD duration of 45 months, were included in this study. The demographic data and clinical characteristics of all participants are shown in Table 2. Among them, 86 (46.2%) patients were men, 75 (40.3%) had DM, 145 (78.0%) had hypertension, and 75 (40.3%) had hyperlipidemia. Female patients had more continuous ambulatory peritoneal dialysis (CAPD) use (*p* = 0.050) and had lower weight (*p* < 0.001), BMI (*p* = 0.009), WC (*p* < 0.001), MAMC (*p* < 0.001), CC (*p* < 0.001), ASMI (*p* < 0.001), and HGS (*p* < 0.001) but higher FTI (*p* = 0.041) than male patients. Regarding laboratory data, female patients showed higher Kt/V (*p* < 0.001) and nPNA (*p* = 0.008) but lower BUN (*p* = 0.018), Cr (*p* < 0.001), phosphorus (*p* = 0.027), and total Cr excretion (*p* < 0.001) levels than male patients. No significant differences in SARC-F and SARC-CalF were observed between genders. 

**Figure 1:**
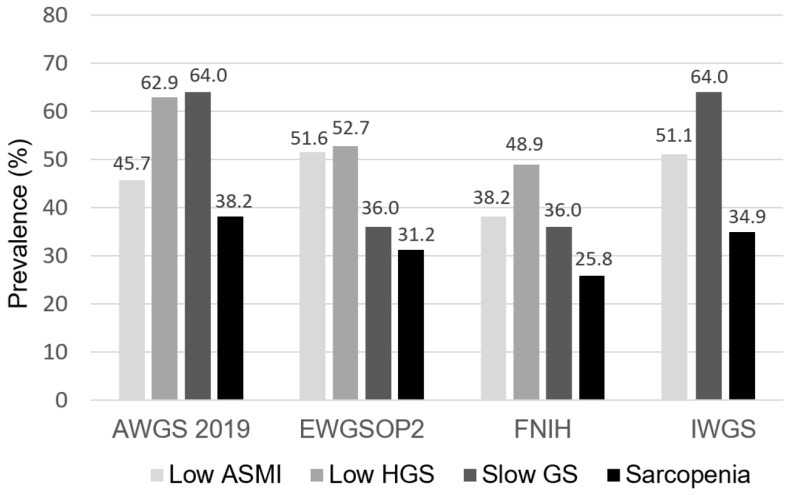
The prevalence of low ASMI, low HGS, slow GS, and sarcopenia across four sarcopenia criteria among patients undergoing PD. ASMI, appendicular skeletal muscle index; HGS, handgrip strength; GS, gait speed; AWGS, Asian Working Group for Sarcopenia; EWGSOP, European Working Group on Sarcopenia in Older People; FNIH, Foundation for the National Institutes of Health; IWGS, International Working Group on Sarcopenia.

The prevalence of low ASMI, low HGS, slow GS, and sarcopenia across the four sarcopenia criteria is illustrated in Figure 1. The prevalence rates of sarcopenia among patients undergoing PD were 38.2%, 31.2%, 25.8%, and 34.9% using the AWGS 2019, EWGSOP2, FNIH, and IWGS, respectively (Figure 1).

The correlations of SARC-F, SARC-CalF, and CC with anthropometric and skeletal muscle measurements are shown in Table 3. SARC-F correlated significantly with HGS (*r* = −0.363, *p* < 0.001) and GS (*r* = −0.452, *p* < 0.001) but not with ASMI (*r* = −0.125, *p* = 0.090) and anthropometric measurements. In contrast, SARC-CalF and CC correlated not only with HGS (*r* = −0.445, *p* < 0.001 for SARC-CalF; *r* = 0.522, *p* < 0.001 for CC) and GS (*r* = −0.293, *p* < 0.001 for SARC-CalF; *r* = 0.181, *p* = 0.019 for CC) but also with ASMI (*r* = −0.421, *p* < 0.001 for SARC-CalF; *r* = 0.683, *p* < 0.001 for CC).

**Figure 2:**
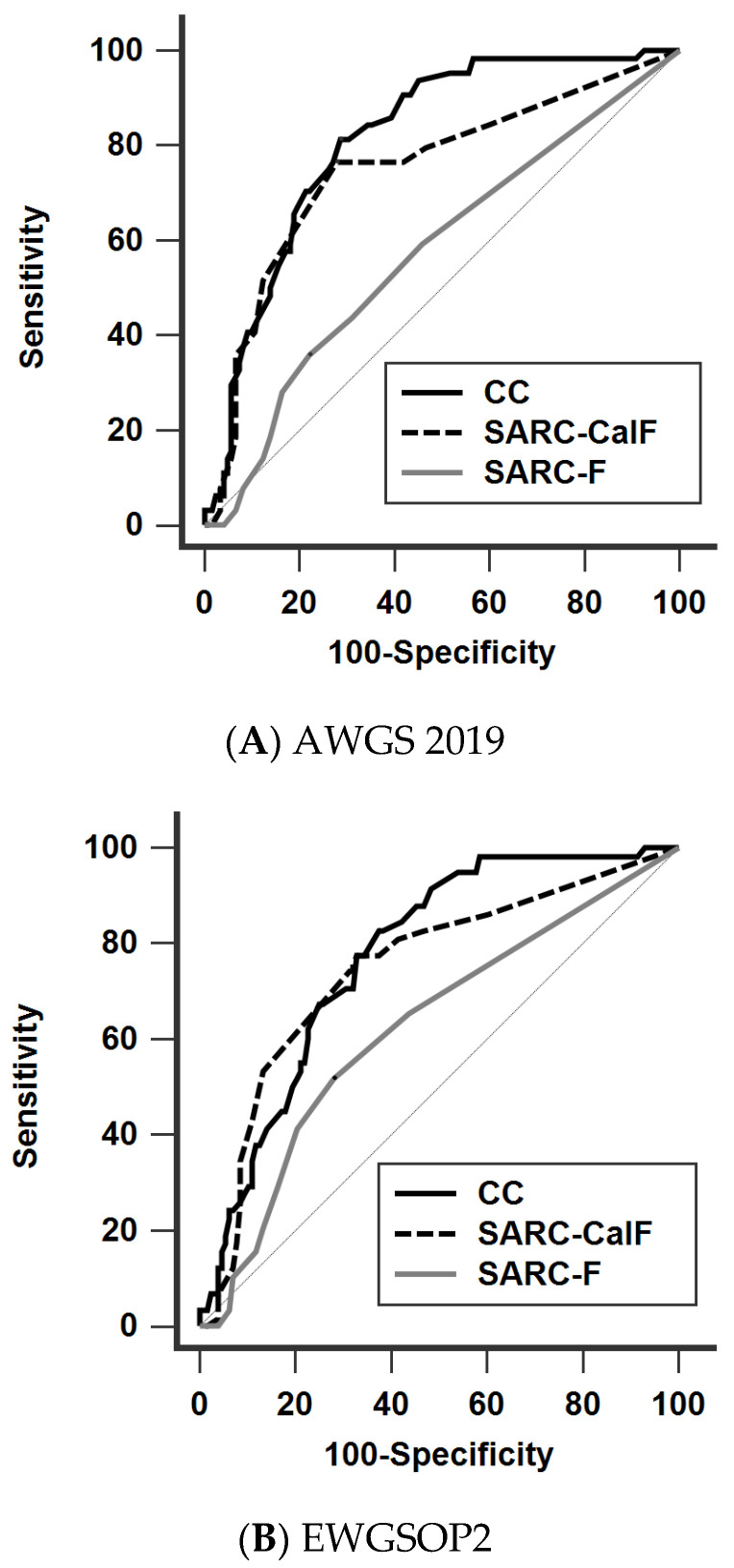

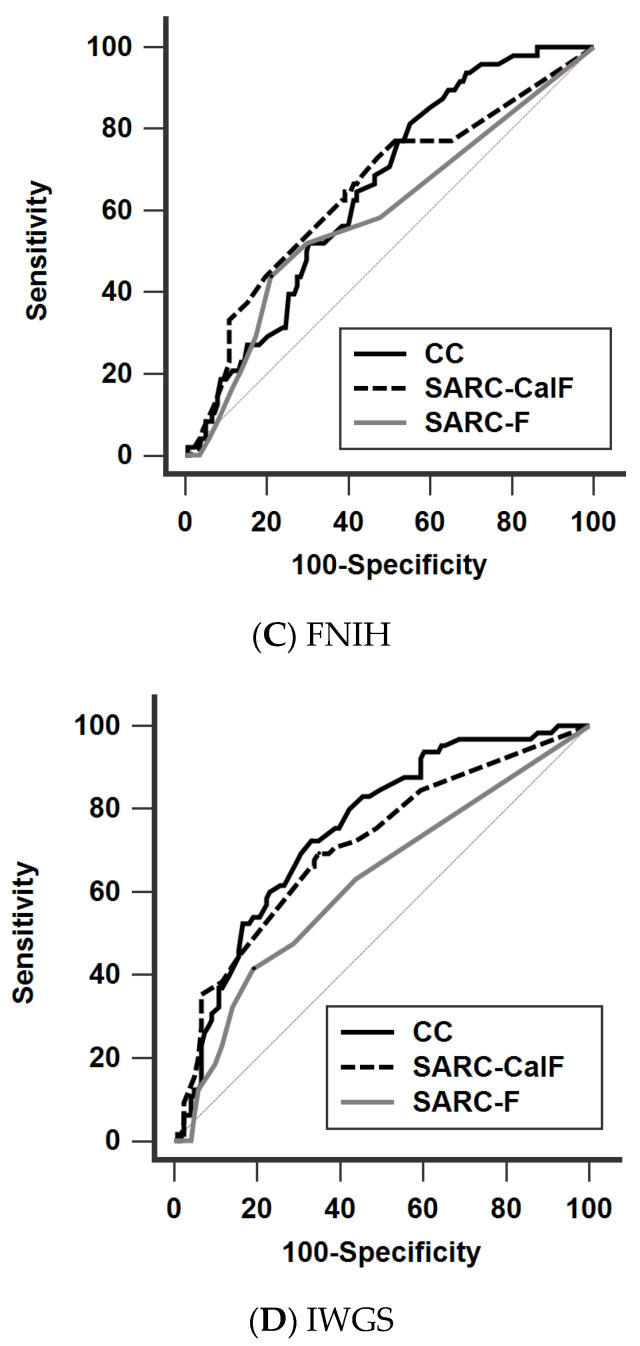
Receiver operating characteristic curves of SARC-F, SARC-CalF, and CC against the four different diagnostic criteria (**A**) AWGS 2019, (**B**) EWGSOP2, (**C**) FNIH, and (**D**) IWGS. AWGS, Asian Working Group for Sarcopenia; EWGSOP, European Working Group on Sarcopenia in Older People; FNIH, Foundation for the National Institutes of Health; IWGS, International Working Group on Sarcopenia, CC, calf circumference; SARC-F, strength, assistance walking, rise from a chair, climb stairs, and falls; SARC-CalF, SARC-F combined with calf circumference.

The diagnostic performance of SARC-F, SARC-CalF, and CC against the four different definitions is shown in Figure 2 (Figure 2A: AWGS 2019, Figure 2B: EWGSOP2, Figure 2C: FNIH, Figure 2D: IWGS) and Table 4. In general, the AUCs of CC (range 0.652–0.813) and SARC-CalF (range 0.648–0.748) were significantly higher than those of SARC-F (range 0.587–0.625) across the different definitions, except when applying FNIH. Furthermore, CC significantly outperformed SARC-CalF when AWGS 2019 was adopted. The trend of the diagnostic performance remained unchanged in our subgroup analysis, stratified by age and PD duration (Supplementary Materials, Tables S1 and S2).

The AUCs of SGA on sarcopenia prediction against the four definitions were also analyzed, which ranged between 0.615 and 0.689.

The AUCs, cut-off values, sensitivity, specificity, PPV, and NPV after stratification by gender among these sarcopenia screening tools are shown in Table 5 and Table 6. In men, the AUC ranges of CC, SARC-CalF, and SARC-F were 0.749–0.863, 0.650–0.813, and 0.495–0.624, respectively; in women, the respective AUC ranges were 0.654–0.784, 0.688–0.708, and 0.590–0.745. The superiority of SARC-CalF and CC over SARC-F was sustained after stratification, except when applying FNIH in women.

## 4. Discussion

To the best of our knowledge, this is the first study to evaluate and compare the diagnostic performance of SARC-F, SARC-CalF, and CC in sarcopenia screening among patients undergoing PD. Our findings suggest that CC and SARC-CalF improved the overall accuracy and low sensitivity of the original SARC-F among patients undergoing PD, irrespective of whether the AWGS 2019, EWGSOP2, or IWGS was applied.

Although sarcopenia is highly prevalent among patients with ESRD, ranging from 11% to 40% [27,28,29,30,31], there has been no consensus regarding the definition and working diagnosis of sarcopenia in patients undergoing dialysis. In an attempt to better characterize the performance of these screening tools, four different operational diagnoses derived from the agreements of geriatric experts were used simultaneously in our study. The prevalence of sarcopenia among our participants undergoing PD ranged between 25.8% and 38.2%. 

Given the high burden of sarcopenia among patients with ESRD, it is crucial to develop simple screening tools for sarcopenia in this population. In patients undergoing HD, Yamamoto et al. have reported that the AUCs of the SARC-F questionnaire for muscle weakness and poor physical performance range from 0.76 to 0.87, indicating its good diagnostic performance for identifying patients undergoing HD with physical disability [32]. Furthermore, a close relationship between SARC-F scores and overall mortality in patients undergoing HD has been demonstrated in our previous study [33]. Unfortunately, the AUCs of SARC-F for sarcopenia, defined as both low muscle mass and strength, were less satisfactory in geriatric and dialysis populations [33,34]. Similarly, in our PD cohort, the diagnostic performance of SARC-F on sarcopenia was generally poor across the four different criteria.

In patients undergoing HD, Marini et al. have reported that SARC-F is more closely associated with muscle functionality than muscle mass [35]; similarly, we disclosed a poor correlation of SARC-F with skeletal muscle mass, including MAMC and ASMI, in patients undergoing PD. These findings suggest that the score of SARC-F primarily reflected the status of skeletal muscle strength and physical performance rather than muscle mass, the latter of which is considered as an essential criterion for sarcopenia diagnosis. In contrast, CC yielded the highest correlation with ASMI in our analysis. In this regard, SARC-CalF, which adds the CC item into SARC-F, could improve the weakness of SARC-F in the aspect of skeletal muscle mass assessment. Not surprisingly, the improved performance of SARC-CalF over SARC-F exhibited in our PD cohort had been consistently reported in the geriatric and cancer population [12,13,14,15].

In particular, CC is considered a strong and reliable marker for skeletal muscle mass in the general population, which exhibited a high correlation with appendicular lean mass in a large-scale NHANES 1999–2006 cohort [36]. The good diagnostic performance of CC for detecting sarcopenia was affirmed in middle-aged and older adults [9,10,11,37] and in patients with chronic liver disease [38] and stroke [39]. In our patients undergoing PD, CC yielded the best correlation not only with ASMI but also with HGS among the three screening tools. The discriminative power of CC was even significantly better than that of SARC-CalF when we adopted the AWGS 2019—the criteria that may be most suitable for our Taiwanese population over the other three definitions. When applying this criterion, the best cut-off of CC was ≤34 cm in males, which provided 90.3% sensitivity, 70.9% specificity, 63.6% PPV, and 92.9% NPV; in females, the best cut-off was ≤33 cm, which provided 82.5% sensitivity, 61.7% specificity, 58.9% PPV, and 84.1% NPV. These findings emphasize that CC could be a simple-to-measure and valuable tool for the initial screening of sarcopenia among patients undergoing PD. 

Although the SGA is a well-validated and widely used nutritional questionnaire to evaluate general nutritional status and predicts clinical outcomes in patients undergoing dialysis, CC or SARC-CalF appeared to be more specific in the assessment of skeletal muscle health in our study. Therefore, incorporating SARC-CalF or CC, together with SGA, into routine clinical practice may provide a more comprehensive evaluation of both nutritional and skeletal muscle status among patients undergoing PD. 

Notably, the diagnostic performance of SARC-CalF and CC on sarcopenia screening in females was inferior to that in males, which was also observed in previous studies [9,10]. Greater calf subcutaneous fat accumulation in females might explain the weaker correlation of CC with skeletal muscle mass [40].

Although this is the first investigation to compare the diagnostic performance of SARC-F, SARC-CalF, and CC in sarcopenia screening among patients undergoing PD, the results of our analysis should be interpreted in the context of several limitations. First, the gold standard for skeletal muscle mass assessment, such as dual-energy X-ray absorptiometry, computed tomography, and magnetic resonance imaging, was not available in this study. Second, hydration status could overestimate the measurement of CC in patients undergoing dialysis. Third, the impacts of SARC-F, SARC-CalF, and CC on clinical outcomes among patients undergoing PD remain undetermined and need to be evaluated in further studies. Fourth, the advantage of CC might be attenuated by obese status, as indicated by the observation from the geriatric population [41]. Fifth, information about physical activity was not available in this study. Finally, this study was conducted in Taiwan, and the cut-off values should be extrapolated with caution, especially to other non-Asian ethnic groups.

## 5. Conclusions

We conclude that among the widely used screening tools for sarcopenia, CC and SARC-CalF outperformed SARC-F in the diagnostic accuracy of sarcopenia among patients undergoing PD, and both could serve as optimal screening tools for sarcopenia in clinical settings.

## Figures and Tables

**Table 1:** Classifications and cut-off values to define sarcopenia in this study.

Classification	AWGS 2019	EWGSOP2	FNIH	IWGS
Low ASMI				
Male	ASM/height^2^ < 7.0 kg/m^2^	ASM/height^2^ < 7.0 kg/m^2^	ASM/BMI < 0.789	ASM/height^2^ < 7.23 kg/m^2^
Female	ASM/height^2^ < 5.7 kg/m^2^	ASM/height^2^ < 6.0 kg/m^2^	ASM/BMI < 0.512	ASM/height^2^ < 5.67 kg/m^2^
Low HGS				
Male	<28 kg	<27 kg	<26 kg	—
Female	<18 kg	<16 kg	<16 kg	—
Slow GS	<1.0 m/s	≤0.8 m/s	≤0.8 m/s	<1.0 m/s
Diagnosis	Low ASMI plus low HGS or slow GS	Low ASMI and low HGS	Low ASMI and low HGS	Low ASMI and slow GS

AWGS, Asian Working Group for Sarcopenia; EWGSOP, European Working Group on Sarcopenia in Older People; FNIH, Foundation for the National Institutes of Health; IWGS, International Working Group on Sarcopenia; ASMI, appendicular skeletal muscle index; BMI, body mass index; HGS, handgrip strength; GS, gait speed.

**Table 2:** Clinical characteristics of the 186 patients undergoing peritoneal dialysis.

Characteristics	All Patients (*n* = 186)	Men (*n* = 86)	Women (*n* = 100)	*p*
Demographics				
Age (years)	57.5 ± 14.1	57.4 ± 13.8	57.6 ± 14.5	0.924
PD duration (months)	45 (19–76)	53 (21–73)	36 (17–83)	0.384
Modality, *n* (%)				
CAPD	97 (52.2)	37 (43.0)	60 (60.0)	0.050 *
APD	85 (45.7)	46 (53.5)	39 (39.0)	
CCPD	4 (2.2)	3 (3.5)	1 (1.0)	
Current smoking, *n* (%)	0 (0)	0 (0)	0 (0)	–
Diseases				
DM, *n* (%)	75 (40.3)	36 (41.9)	39 (39.0)	0.692
Hypertension, *n* (%)	145 (78.0)	69 (80.2%)	76 (76.0)	0.488
Hyperlipidemia, *n* (%)	75 (40.3)	33 (38.4)	42 (42.0)	0.615
Examination				
Weight (kg)	64.9 ± 13.7	72.2 ± 12.4	58.6 ± 11.4	<0.001 *
BMI (kg/m^2^)	25.0 ± 4.1	25.9 ± 3.5	24.3 ± 4.4	0.009 *
WC (cm)	92.5 ± 10.3	96.1 ± 9.2	89.4 ± 10.2	<0.001 *
MAMC (cm)	23.8 ± 3.1	25.3 ± 2.5	22.6 ± 3.0	<0.001 *
CC (cm)	34 ± 4	35 ± 4	33 ± 4	<0.001 *
FTI (kg/m^2^)	11.1 ± 4.6	10.3 ± 4.1	11.7 ± 4.9	0.041 *
ASMI (kg/m^2^)	6.6 ± 1.3	7.3 ± 1.1	5.8 ± 1.1	<0.001 *
HGS (kg)	22.2 ± 8.5	27.0 ± 7.6	16.6 ± 6.3	<0.001 *
GS (m/s)	0.9 ± 0.3	0.9 ± 0.2	0.9 ± 0.3	0.108
Questionnaire				
SGA score	11 (9–12)	11 (9–12)	11 (9–12)	0.989
SARC-F	1 (0–3)	0 (0–2)	1 (0–3)	0.165
SARC-CalF	4 (0–11)	2 (0–10)	9 (0–12)	0.069
Laboratory data				
Kt/V	2.0 (1.7–2.2)	1.8 (1.6–2.1)	2.0 (1.7–2.4)	<0.001 *
Hemoglobin (g/dL)	9.7 (8.7–10.8)	9.9 (8.6–10.6)	9.7 (8.9–10.5)	0.338
Albumin (g/dL)	3.6 (3.3–3.8)	3.7 (3.3–3.9)	3.6 (3.3–3.8)	0.197
BUN (mg/dL)	61 (50–75)	64 (53–76)	58 (46–73)	0.018 *
Cr (mg/dL)	10.8 ± 3.0	12.1 ± 3.2	9.8 ± 2.4	<0.001 *
Phosphorus (mg/dL)	5.1 (4.4–6.0)	5.2 (4.5–6.4)	4.9 (4.3–5.7)	0.027 *
Intact PTH (pg/mL)	245 (96–494)	277 (110–546)	185 (90–426)	0.148
nPNA (g/kg/day)	0.91 (0.79–1.09)	0.87 (0.77–1.01)	0.96 (0.82–1.13)	0.008 *
Total Cr excretion (mg/day)	931 (694–1178)	1102 (882–1318)	862 (628–1010)	<0.001 *

PD, peritoneal dialysis; CAPD, continuous ambulatory peritoneal dialysis; APD, automated peritoneal dialysis; CCPD, continuous cycling peritoneal dialysis; DM, diabetes mellitus; BMI, body mass index; WC, waist circumference; MAMC, mid-arm muscular circumference; CC, calf circumference; FTI, fat tissue index; ASMI, appendicular skeletal muscle index; HGS, handgrip strength; GS, gait speed; SGA, subjective global assessment; SARC-F, strength, assistance walking, rise from a chair, climb stairs, and falls; SARC-CalF, SARC-F combined with calf circumference, Kt/V, fractional clearance index for urea; BUN, blood urea nitrogen; Cr, creatinine; PTH, parathyroid hormone; nPNA, normalized protein nitrogen appearance. * *p* < 0.05 was considered to be statistically significant, comparing differences between males and females.

**Table 3:** The correlations of SARC-F, SARC-CalF, and CC with anthropometric and skeletal muscle measurements.

Variables	SARC-F		SARC-CalF		CC	
	*r*	*p*	*r*	*p*	*r*	*p*
Anthropometric measures						
Weight (kg)	−0.029	0.692	−0.435	<0.001 *	0.721	<0.001 *
BMI (kg/m^2^)	−0.009	0.900	−0.382	<0.001 *	0.625	<0.001 *
WC (cm)	0.120	0.104	−0.224	0.002 *	0.436	<0.001 *
MAMC (cm)	−0.056	0.451	−0.395	<0.001 *	0.617	<0.001 *
FTI (kg/m^2^)	0.136	0.067	−0.146	0.050 *	0.298	<0.001 *
Skeletal muscle measures						
ASMI (kg/m^2^)	−0.125	0.090	−0.421	<0.001 *	0.683	<0.001 *
HGS (kg)	−0.363	<0.001 *	−0.445	<0.001 *	0.522	<0.001 *
GS (m/s) ^a^	−0.452	<0.001 *	−0.293	<0.001 *	0.181	0.019 *

^a^ *n* = 168, CC, calf circumference; BMI, body mass index; WC, waist circumference; MAMC, mid-arm muscular circumference; FTI, fat tissue index; ASMI, appendicular skeletal muscle index; HGS, handgrip strength; GS, gait speed. * *p* < 0.05 was considered to be statistically significant.

**Table 4:** The diagnostic performance of SARC-F, SARC-CalF, and CC on sarcopenia based on four operational definitions in the overall study population.

Definitions	AUC (95% CI)	*p*
AWGS 2019		
CC	0.813 (0.749–0.866) ^a,b^	<0.001 *
SARC-CalF	0.739 (0.670–0.801) ^a,c^	<0.001 *
SARC-F	0.587 (0.513–0.659) ^b,c^	0.033 *
EWGSOP2		
CC	0.776 (0.709–0.834) ^b^	<0.001 *
SARC-CalF	0.748 (0.679–0.809) ^c^	<0.001 *
SARC-F	0.625 (0.551–0.695) ^b,c^	0.003 *
FNIH		
CC	0.652 (0.579–0.721)	<0.001 *
SARC-CalF	0.648 (0.575–0.717)	0.002 *
SARC-F	0.587 (0.513–0.659)	0.063
IWGS		
CC	0.750 (0.682–0.811) ^b^	<0.001 *
SARC-CalF	0.710 (0.639–0.774) ^c^	<0.001 *
SARC-F	0.621 (0.547–0.691) ^b,c^	0.004 *

AUC, area under curve; CI, confidence interval; AWGS, Asian Working Group for Sarcopenia; CC, calf circumference; SARC-F, strength, assistance walking, rise from a chair, climb stairs, and falls; SARC-CalF, SARC-F combined with calf circumference; EWGSOP, European Working Group on Sarcopenia in Older People; FNIH, Foundation for the National Institutes of Health; IWGS, International Working Group on Sarcopenia. ^a^ *p* < 0.05 indicates significant difference of AUCs between CC and SARC-CalF. ^b^ *p* < 0.05 indicates significant difference of AUCs between CC and SARC-F. ^c^ *p* < 0.05 indicates significant difference of AUCs between SARC-CalF and SARC-F. * The AUC was significantly different from 0.5.

**Table 5:** The diagnostic performance of SARC-F, SARC-CalF, and CC on sarcopenia based on four operational definitions in male patients undergoing PD.

Definitions	AUC (95% CI)	Cut-Off	Sen (%)	Spe (%)	PPV (%)	NPV (%)
AWGS 2019						
CC	0.859 (0.768–0.925) *	≤34	90.3	70.9	63.6	92.9
SARC-CalF	0.804 (0.704–0.881) *	≥10	74.2	81.8	69.7	84.9
SARC-F	0.588 (0.477–0.693)	≥1	58.1	60.0	45.0	71.7
EWGSOP2						
CC	0.863 (0.772–0.927) *	≤34	82.1	81.0	67.6	90.4
SARC-CalF	0.813 (0.714–0.889) *	≥10	78.6	81.0	66.7	88.7
SARC-F	0.586 (0.475–0.691)	≥1	57.1	58.6	40.0	73.9
FNIH						
CC	0.749 (0.644–0.836) *	≤36	96.7	46.4	49.2	96.3
SARC-CalF	0.650 (0.539–0.749) *	≥10	60.0	73.2	54.5	77.4
SARC-F	0.495 (0.385–0.605)	≥3	26.7	80.4	32.8	57.9
IWGS						
CC	0.792 (0.691–0.872) *	≤34	67.7	78.9	67.6	78.8
SARC-CalF	0.746 (0.640–0.834) *	≥10	61.8	76.9	63.6	75.5
SARC-F	0.624 (0.513–0.727) *	≥1	61.8	63.5	52.5	71.7

AUC, area under curve; CI, confidence interval; Sen, sensitivity; Spe, specificity; PPV, positive predictive value; NPV, negative predictive value; AWGS, Asian Working Group for Sarcopenia; CC, calf circumference; EWGSOP, European Working Group on Sarcopenia in Older People; FNIH, Foundation for the National Institutes of Health; IWGS, International Working Group on Sarcopenia; CC, calf circumference; SARC-F, strength, assistance walking, rise from a chair, climb stairs, and falls; SARC-CalF, SARC-F combined with calf circumference. * *p* < 0.05 indicates that the AUC was significantly different from 0.5.

**Table 6:** The diagnostic performance of SARC-F, SARC-CalF, and CC on sarcopenia based on four operational definitions in female patients undergoing PD.

Definitions	AUC (95% CI)	Cut-Off	Sen (%)	Spe (%)	PPV (%)	NPV (%)
AWGS 2019						
CC	0.784 (0.691–0.860) *	≤33	82.5	61.7	58.9	84.1
SARC-CalF	0.688 (0.588–0.777) *	≥7	75.0	61.7	56.6	78.7
SARC-F	0.590 (0.487–0.687)	≥3	47.5	80.0	61.3	69.6
EWGSOP2						
CC	0.726 (0.627–0.810) *	≤31	66.7	68.6	47.6	82.8
SARC-CalF	0.702 (0.602–0.789) *	≥11	56.7	78.6	53.1	80.9
SARC-F	0.665 (0.564–0.757) *	≥2	63.3	70.0	47.5	81.7
FNIH						
SARC-F	0.745 (0.648–0.827) *	≥2	83.3	69.5	37.5	95.0
SARC-CalF	0.708 (0.609–0.795) *	≥13	55.6	86.6	47.6	89.9
CC	0.654 (0.553–0.747) *	≤32	66.7	63.4	28.6	89.7
IWGS						
CC	0.765 (0.669–0.844) *	≤32	71.0	75.4	56.4	85.2
SARC-CalF	0.700 (0.600–0.787) *	≥7	77.4	58.0	45.3	85.1
SARC-F	0.637 (0.534–0.730) *	≥3	54.8	79.7	54.8	79.7

AUC, area under curve; CI, confidence interval; Sen, sensitivity; Spe, specificity; PPV, positive predictive value; NPV, negative predictive value; AWGS, Asian Working Group for Sarcopenia; CC, calf circumference; EWGSOP, European Working Group on Sarcopenia in Older People; FNIH, Foundation for the National Institutes of Health; IWGS, International Working Group on Sarcopenia; CC, calf circumference; SARC-F, strength, assistance walking, rise from a chair, climb stairs, and falls; SARC-CalF, SARC-F combined with calf circumference. * *p* < 0.05 indicates that the AUC was significantly different from 0.5.

## Supplementary Materials

The following supporting information can be downloaded at: https://www.mdpi.com/article/10.3390/nu14050923/s1, Table S1: The diagnostic performance of SARC-F, SARC-CalF, and CC on sarcopenia based on four operational definitions, stratified by age (≥60 or <60 years), Table S2: The diagnostic performance of SARC-F, SARC-CalF, and CC on sarcopenia based on four operational definitions, stratified by PD duration (≥45 or <45 months).
