# Supplementary material for: A Comparison of SARC-F, Calf Circumference, and Their Combination for Sarcopenia Screening among Patients Undergoing Peritoneal Dialysis
Source: Nutrients. 2022 Feb 22;14(5):923. doi: 10.3390/nu14050923 (PMC8912378; doi:10.3390/nu14050923)
Supplement: Supplementary file 1 [file nutrients-14-00923-s001.zip › nutrients-1613878-supplementary.pdf]

Table S1. The diagnostic performance of SARC-F, SARC-CalF, and CC on sarcopenia based on four operational definitions, stratified by age (≥60 or <60 years).

**Definitions**	**AUC (95% CI)**	
	**Age ≥60 years (n = 82)**	**Age <60 years (n = 104)**
AWGS 2019		
CC	0.816 (0.715–0.893)[b]	0.800 (0.710–0.872)[a,b]
SARC-CalF	0.756 (0.649–0.844)[c]	0.680 (0.581–0.768)[a,c]
SARC-F	0.598 (0.484–0.705)[b,c]	0.518 (0.418–0.617)[b,c]
EWGSOP2		
CC	0.765 (0.659–0.852)[b]	0.762 (0.669–0.840)
SARC-CalF	0.727 (0.618–0.820)[c]	0.714 (0.617–0.799)
SARC-F	0.581 (0.466–0.689)[b,c]	0.613 (0.512–0.707)
FNIH		
CC	0.577 (0.463–0.686)	0.689 (0.591–0.776)
SARC-CalF	0.546 (0.432–0.657)	0.726 (0.630–0.809)
SARC-F	0.531 (0.417–0.642)	0.584 (0.483–0.680)
IWGS		
CC	0.832 (0.733–0.905)[b]	0.642 (0.543–0.734)[a]
SARC-CalF	0.799 (0.696–0.880)[c]	0.536 (0.436–0.634)[a]
SARC-F	0.638 (0.525–0.742)[b,c]	0.533 (0.433–0.632)

AUC, area under curve; CI, confidence interval; CC, calf circumference; SARC-F, strength, assistance walking, rise from a chair, climb stairs, and falls; SARC-CalF, SARC-F combined with calf circumference; AWGS, Asian Working Group for Sarcopenia; CC, calf circumference; EWGSOP, European Working Group on Sarcopenia in Older People; FNIH, Foundation for the National Institutes of Health; IWGS, International Working Group on Sarcopenia. [a] $p < 0.05$ indicates significant difference of AUCs between CC and SARC-CalF. [b] $p < 0.05$ indicates significant difference of AUCs between CC and SARC-F. [c] $p < 0.05$ indicates significant difference of AUCs between SARC-CalF and SARC-F.

Table S2. The diagnostic performance of SARC-F, SARC-CalF, and CC on sarcopenia based on four operational definitions, stratified by PD duration (≥45 or <45 months).

Definitions	AUC (95% CI)	
	**PD duration ≥45 months (*n* = 93)**	**PD duration <45 months (*n* = 93)**
AWGS 2019		
CC	0.831 (0.738–0.901)[b]	0.793 (0.697–0.869)[a,b]
SARC-CalF	0.765 (0.665–0.847)[c]	0.713 (0.610–0.802)[a,c]
SARC-F	0.577 (0.469–0.679)[b,c]	0.596 (0.490–0.696)[b,c]
EWGSOP2		
CC	0.803 (0.707–0.878)[b]	0.747 (0.647–0.831)
SARC-CalF	0.775 (0.676–0.856)[c]	0.720 (0.618–0.808)
SARC-F	0.613 (0.505–0.712)[b,c]	0.636 (0.530–0.733)
FNIH		
CC	0.708 (0.604–0.798)[b]	0.584 (0.478–0.685)
SARC-CalF	0.683 (0.578–0.776)[c]	0.607 (0.501–0.706)
SARC-F	0.566 (0.458–0.669) [b,c]	0.604 (0.498–0.703)
IWGS		
CC	0.760 (0.660–0.843)	0.740 (0.639–0.825)
SARC-CalF	0.726 (0.623–0.814)[c]	0.689 (0.585–0.781)
SARC-F	0.629 (0.522–0.727)[c]	0.605 (0.499–0.704)

PD, peritoneal dialysis; AUC, area under curve; CI, confidence interval; CC, calf circumference; SARC-F, strength, assistance walking, rise from a chair, climb stairs, and falls; SARC-CalF, SARC-F combined with calf circumference; AWGS, Asian Working Group for Sarcopenia; CC, calf circumference; EWGSOP, European Working Group on Sarcopenia in Older People; FNIH, Foundation for the National Institutes of Health; IWGS, International Working Group on Sarcopenia. [a] $p < 0.05$ indicates significant difference of AUCs between CC and SARC-CalF. [b] $p < 0.05$ indicates significant difference of AUCs between CC and SARC-F. [c] $p < 0.05$ indicates significant difference of AUCs between SARC-CalF and SARC-F.
